# Recipient-Related Clinical Risk Factors for Primary Graft Dysfunction after Lung Transplantation: A Systematic Review and Meta-Analysis

**DOI:** 10.1371/journal.pone.0092773

**Published:** 2014-03-21

**Authors:** Yao Liu, Yi Liu, Lili Su, Shu-juan Jiang

**Affiliations:** Department of Respiratory Medicine, Provincial Hospital Affiliated to Shandong University, Jinan, Shandong, China; University of Pittsburgh, United States of America

## Abstract

**Background:**

Primary graft dysfunction (PGD) is the main cause of early morbidity and mortality after lung transplantation. Previous studies have yielded conflicting results for PGD risk factors. Herein, we carried out a systematic review and meta-analysis of published literature to identify recipient-related clinical risk factors associated with PGD development.

**Method:**

A systematic search of electronic databases (PubMed, Embase, Web of Science, Cochrane CENTRAL, and Scopus) for studies published from 1970 to 2013 was performed. Cohort, case-control, or cross-sectional studies that examined recipient-related risk factors of PGD were included. The odds ratios (ORs) or mean differences (MDs) were calculated using random-effects models

**Result:**

Thirteen studies involving 10042 recipients met final inclusion criteria. From the pooled analyses, female gender (OR 1.38, 95% CI 1.09 to 1.75), African American (OR 1.82, 95%CI 1.36 to 2.45), idiopathic pulmonary fibrosis (IPF) (OR 1.78, 95% CI 1.49 to 2.13), sarcoidosis (OR 4.25, 95% CI 1.09 to 16.52), primary pulmonary hypertension (PPH) (OR 3.73, 95%CI 2.16 to 6.46), elevated BMI (BMI≥25 kg/m^2^) (OR 1.83, 95% CI 1.26 to 2.64), and use of cardiopulmonary bypass (CPB) (OR 2.29, 95%CI 1.43 to 3.65) were significantly associated with increased risk of PGD. Age, cystic fibrosis, secondary pulmonary hypertension (SPH), intra-operative inhaled nitric oxide (NO), or lung transplant type (single or bilateral) were not significantly associated with PGD development (all *P*>0.05). Moreover, a nearly 4 fold increased risk of short-term mortality was observed in patients with PGD (OR 3.95, 95% CI 2.80 to 5.57).

**Conclusions:**

Our analysis identified several recipient related risk factors for development of PGD. The identification of higher-risk recipients and further research into the underlying mechanisms may lead to selective therapies aimed at reducing this reperfusion injury.

## Introduction

Although lung transplantation has become an increasingly common procedure in recent years, it has consistently lagged behind other organs in survival rates [1], and early postoperative allograft dysfunction remains a significant cause of post-transplantation morbidity and mortality [2]. Primary graft dysfunction (PGD) is a severe form of acute lung injury induced by ischemia-reperfusion injury that occurs in approximately 10–25% of lung graft recipients [2], [3]. Reported 30-day mortality rates of patients with severe PGD are nearly 8 times as high as those for patients without PGD [4]. PGD leads to increased duration of mechanical ventilation and intensive care unit stay, poor functional outcomes, and increase rates of perioperative complications [5].

A number of previous studies have been designed to identify the clinical risk factors associated with PGD [6]–[23]. This field is of great clinical interest, since better understanding those transplant recipients most at risk might revolve around a concept of earlier detection for targeted therapy and aggressive support. In this regard, a number of clinical risk factors have been identified, including both organ donor and recipient characteristics. Donor characteristics previously identified include female gender, African American race, heavy smokers, older (>45 yr) or younger (<21 yr) donor age, and closed head injury as a cause of death [9], [10], [18], [19], [20]. Recipient characteristics previously linked to PGD include a diagnosis of primary pulmonary hypertension (PPH) [6], [10], [13], [19], and elevated pulmonary artery pressures (PAP) [6], [19]. In spite of this, there are several recipient-related risk factors that have been inconsistently reported in the literature.

Considering a single study may lack the power of providing a reliable conclusion, we carried out a rigorous systematic review and meta-analysis of published literature to gain more precise and quantitative estimates of recipient-related risk factors associated with development of PGD.

## Methods

This meta-analysis followed the Meta-analysis of Observational Studies in Epidemiology (MOOSE) guidelines [24].

### Search Strategy

Two reviewers (YL and SJJ) systematically searched PubMed, Embase, ISI Web of Science, Cochrane CENTRAL, and Scopus for articles published until October 2013. The following keywords were used in searching: “primary graft dysfunction” or “primary graft failure” or “ischemia-reperfusion injury” or “acute lung injury” or “early graft failure”, combined with “lung transplantation”. Language restrictions were not applied. From the title, abstract or descriptors, the literature search was reviewed independently to identify potentially relevant trials for full review. The “related articles” function was used to broaden the search. In addition, a manual review of references from primary or review articles was performed to identify any additional relevant studies.

### Study selection

Cohort, case-control, and cross-sectional studies were included if they investigated which recipient-related factors directly influencing the development of PGD after lung transplantation. The potential variables assessed could be recipient demographics, co-morbidities, laboratory test, operative data, and postoperative complications. We did not address molecular or genetic markers as these require access to laboratory resources and genetic expertise. After obtaining full reports of candidate studies, the same reviewers independently assessed eligibility. Differences in data between the two reviewers were resolved by reviewing corresponding articles, and the final set was agreed on by consensus. When multiple articles for a single study had been published, we used the latest publication and supplemented it, if necessary, with data from the earlier publications. Attempts were also made to contact investigators for unpublished data.

### Data Extraction

Two investigators (YL and SJJ) independently summarized the studies meeting the inclusion criteria, and performed data extraction using a standard data sheet [25]. Disagreement was resolved by consensus or by a third party. For each study, the following data were extracted: first author's last name, publication year, study date, country, study design, sample size, patient characteristics (age and gender) and definition of PGD. Initially, we scrutinized in detail the literature about PGD after lung transplantation to identify all possible risk factors. The initial search yielded 18 possible risk factors. Following review by an expert panel (YL, LLS and SJJ), 10 factors that were considered to be easily measured in routine clinical practice and had been analyzed in at least 2 studies were selected for the full systematic review. These factors assessed including age, gender, race, pulmonary diagnosis, PAP, type of transplant (single lung transplant (SLT) vs bilateral lung transplant (BLT)), body mass index (BMI), cardiopulmonary bypass (CPB), intra-operative inhaled nitric oxide (NO), and blood products transfusion.

### Study Quality Assessment

The Newcastle-Ottawa Scale was used to assess the quality of observational studies based on the following nine questions: (1) representativeness of the exposed cohort; (2) selection of the non-exposed cohort; (3) ascertainment of exposure; (4) demonstration that the outcome was not present at outset of study; (5) comparability; (6) assessment of outcome; (7) length of follow-up sufficient; (8) adequacy of participant follow-up; (9) total stars [26]. Maximum score on this scale is a total of 9. “Good” was defined as a total score of 7 to 9; “fair,” a total score of 4–6; and “poor,” defined as a total score of <4.

### Statistical analyses

Our meta-analysis and statistical analyses were performed with Revman software (version 5.2; Cochrane Collaboration, Oxford, United Kingdom) and Stata software (version 11.0; Stata Corporation, College Station, TX, USA). The odds ratios (ORs) and 95% confidence intervals (CIs) were calculated to estimate the association between binary factors and development of PGD. When mean values and SDs for a certain risk factor were provided, we calculated the mean differences (MDs) between patients with and without PGD. The statistical estimates of effect were derived using a random-effects (DerSimonian and Laird) model, which assumes that the true underlying effect varies among included studies, because of the different characteristics of study population, transplantation procedure, and the PGD definitions that were involved in the original trials.

The definitions of PGD may be a potential source of heterogeneity. In order to analyze the heterogeneity associated with different definitions, we performed subgroup analyses by comparing summary results obtained from subsets of studies grouped by “the International Society for Heart and Lung Transplantation (ISHLT) PGD Grading System [28]” or other definitions. Statistical heterogeneity of treatment effects between studies was formally tested with Cochran's χ2 statistics and with significance set at *P*<0.10. The I^2^ statistic was used to quantify heterogeneity. Using accepted guidelines [27], an I^2^ of 0% to 40% was considered to exclude heterogeneity, an I^2^ of 30% to 60% to represent moderate heterogeneity, an I^2^ of 50% to 90% to represent substantial heterogeneity, and an I^2^ of 75% to 100% to represent considerable heterogeneity. Publication bias was assessed with funnel plots and the Begg's test.

## Results

### Literature search and study characteristics

**Figure 1:**
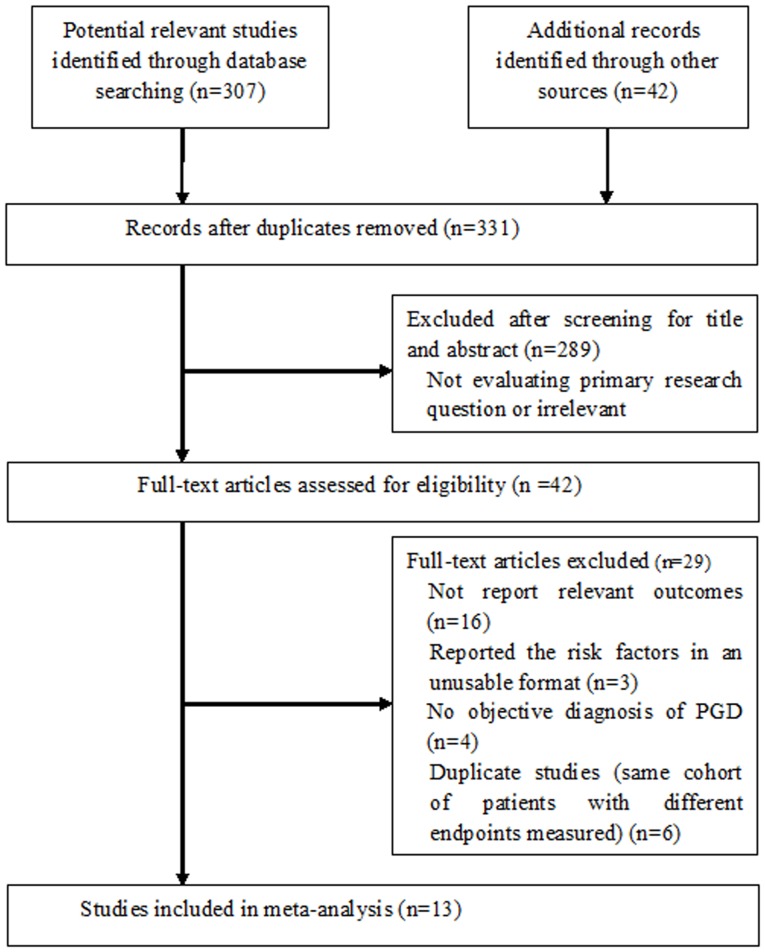
Flow of study identification, inclusion, and exclusion.

The method used to select studies is shown in Figure 1. A total of 331 potentially eligible articles were initially identified, and 289 articles were excluded as they were not relevant to the purpose of the current meta-analysis. Therefore, 42 potentially relevant articles were selected for detailed evaluation. From the overall pool of full-text articles, 29 articles were excluded because they did not provide PGD data according to the risk factors we evaluated (n = 16), reported the risk factors in an unusable format (n = 3), did not make any objective diagnosis of PGD (n = 4), or were duplicate studies (same cohort of patients with different endpoints measured) (n = 6). Thus, 13 studies were included in the meta-analysis with a total of 10042 patients [7]–[19]. Additional data were requested from the authors of three studies but didn't receive any reply.

Baseline characteristics of the studies included are shown in Table 1. The 13 included studies consisted of 5 prospective cohort studies [12], [15]–[17], [19], 7 retrospective analyses of cohort data or chart review [7]–[11], [14], [18], and 1 secondary analysis of multicenter registry [13]. Eight of the 13 studies involved American subjects [7], [9], [10], [13], [15]–[17], [19], and the populations of the remaining five studies came from France [8], [14], Denmark [11], Austria [12], and Brazil [18]. The studies varied in size from 28 to 6984 subjects, and the average age of the patients ranged from 25 to 56 years.

**Table 1:** Characteristics of Selected Studies.

Author	Date of study (Year)	Country	Study Design	No. of Subjects (M/F)	Mean age, %	Definition of PGD	Quality Assessment
King et al,^7^ 2000	1990–1998	USA	Retrospective, single-center chart review	100(NA)	49	Patients with a chest x-ray film (CXR) score of ≥6 and a PaO2/FiO2 gradient of less than 200 mm Hg	Fair
Thabut et al,^8^ 2002	1988–2000	France	Retrospective multicenter cohort study	257 (169/88)	48	The presence of reperfusion pulmonary edema with or without early hemodynamic failure.	Fair
Christie et al,^9^ 2003	199–2000	USA	Retrospective single-center cohort study	252(123/129)	49	The presence of a diffuse alveolar infiltrate and a PaO2/FiO2 gradient of less than 200 mm Hg	Good
Whitson et al,^10^ 2006	1992–2004	USA	Retrospective, single-center chart review	402 (185/217)	50	ISHLT PGD Grading System	Fair
Burton et al,^11^ 2007	1999–2004	Denmark	Retrospective	180 (82/98)	56	The presence of a unilateral diffuse radiological infiltrate of the lung allograft.	Fair
Krenn et al,^12^ 2007	2003–2006	Austria	Prospective single-center cohort study	150 (76/74)	38	ISHLT PGD Grading System	Good
Kuntz et al,^13^ 2009	1994–2002	USA	Secondary analysis of multicenter registry (UNOS/ISHLT)	6984 (4315/2669)	—	A PaO2/FiO2 ratio less than 200, with evidence of radiographic infiltrates, and absence of secondary causes of allograft dysfunction.	Good
Felten et al, ^14^ 2011	2006–2008	France	Retrospective, multicenter cohort study	122 (63/59)	25	ISHLT PGD Grading System	Good
Fang et al,^15^ 2011	2002–2007	USA	Prospective multicenter cohort study	126 (60/66)	56	ISHLT PGD Grading System	Good
Allen et al,^16^ 2012	2002–2007	USA	Prospective, single-center cohort study	28 (12/16)	51	ISHLT PGD Grading System	Fair
Shah et al,^17^ 2012	2006–2008	USA	Prospective multicenter cohort study	108(56/52)	37	ISHLT PGD Grading System	Good
Samano et al,^18^ 2012	2003–2010	Brazil	Retrospective, single-center chart review	78 (46/32)	44	ISHLT PGD Grading System	Fair
Diamond et al,^19^ 2013	2002–2010	USA	Prospective, multicenter cohort study (LTOG)	1255 (211/1044)	35	ISHLT PGD Grading System	Good

M, male; F, female; PGD, primary graft dysfunction; ISHLT, International Society for Heart and Lung Transplantation.

There were some variations in the definition of PGD. The ISHLT PGD grading schema was used in the majority of the studies. The other 5 studies also defined PGD based on the presence of infiltrates in the lung allograft on chest radiograph and/or the PaO2/FiO2 ratio [7]–[9], [11], [13]. PGD, as defined in the original articles, was present in 16.4% of the lung transplant patients. All studies were of high methodological quality (good or fair) as assessed by the Newcastle-Ottawa Scale [26] (Table 1). The risk factors examined in the 13 included studies are summarized in Table 2.

**Table 2:** The recipient-related risk factors examined in the original articles.

Author	Age	Gender	Race	Pulmonary Diagnosis	PAP	BLT vs SLT	BMI	CPB	Inhaled NO	Blood products transfusion	Mortality
King et al,^7^	**√**			**√**	**√**	**√**		**√**			**√**
Thabut et al,^8^	**√**	**√**		**√**		**√**		**√**			**√**
Christie et al,^9^	**√**	**√**	**√**	**√**	**√**	**√**			**√**		
Whitson et al,^10^		**√**		**√**		**√**		**√**			**√**
Burton et al,^11^	**√**	**√**		**√**			**√**	**√**			**√**
Krenn et al,^12^	**√**	**√**		**√**	**√**	**√**		**√**			**√**
Kuntz et al,^13^		**√**	**√**	**√**		**√**	**√**				
Felten et al,^14^	**√**	**√**					**√**	**√**	**√**	**√**	
Fang et al,^15^	**√**	**√**	**√**		**√**	**√**		**√**	**√**	**√**	
Allen et al,^16^	**√**	**√**		**√**	**√**	**√**		**√**	**√**		**√**
Shah et al,^17^	**√**	**√**	**√**	**√**	**√**	**√**		**√**			
Samano et al,^18^	**√**	**√**				**√**		**√**			**√**
Diamond et al,^19^		**√**	**√**	**√**	**√**	**√**	**√**	**√**		**√**	**√**

PAP, pulmonary artery pressure; BLT, bilateral lung transplant; SLT, single lung transplant; BMI, body mass index; CPB, cardiopulmonary bypass; NO, nitric oxide.

### Outcomes and synthesis of results

#### Age

Ten studies investigated the influence of recipient age on the occurrence of PGD [7]–[9], [11], [12], [14]–[18], including 434 patients with PGD and 969 controls. Findings from this analysis suggested no significant difference in mean age between patients with or without PGD (MD -0.75 y, 95% CI -2.12 to 0.63 y, *P* = 0.29). Statistical heterogeneity among the studies was significant (I^2^ = 61%, *P* = 0.006).

#### Gender

**Figure 2:**
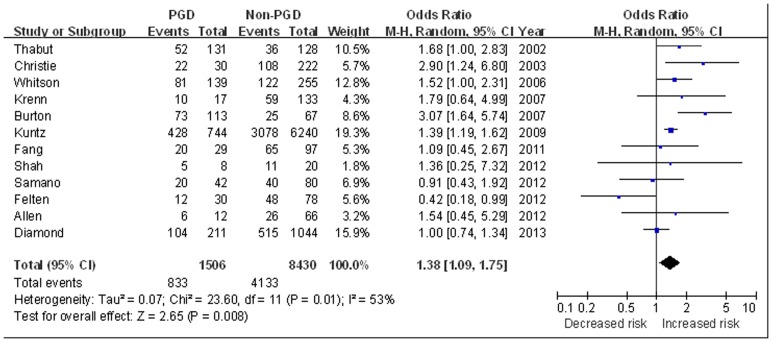
The influence of recipient gender on PGD.

Twelve studies investigated the influence of recipient gender on the occurrence of PGD [8]–[19]. These studies included 1506 patients with PGD and 8430 controls. The proportion of female recipients was 55.3% in patients with PGD compared with 49.0% in patients without. Analysis suggested female recipients had an increased risk of PGD (OR 1.38, 95% CI 1.09 to 1.75, *P* = 0.008) (Figure 2).

#### Race

**Figure 3:**
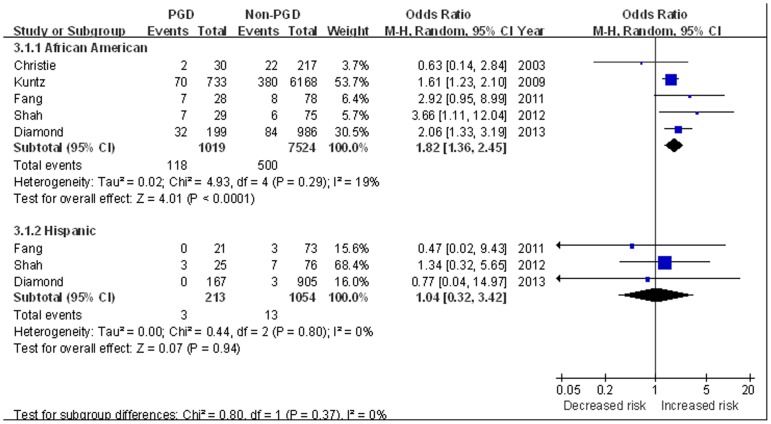
The influence of African American and Hispanic race on PGD compared with white race.

Five studies reported the influence of recipient race [9], [13], [15], [17], [19]. PGD was found in 11.4% of patients with white race, 19.1% of African American patients, and 18.7% of Hispanic patients. White race was used as the reference group given the lowest incidence of PGD. Analysis of these studies showed compared with white race, African American was associated with a significantly increased risk of PGD (OR 1.82, 95%CI 1.36 to 2.45, *P*<0.0001), while Hispanic race did not appear to affect the risk of PGD (OR 1.04, 95%CI 0.32 to 3.42, *P* = 0.94) (Figure 3).

#### Pulmonary Diagnosis

The effect of recipient pulmonary diagnosis on PGD development was evaluated in 10 studies [7]–[13], [16], [17], [19]. The incidence of PGD was 11.8% in patients with chronic obstructive pulmonary disease (COPD), 18.0% in patients with idiopathic pulmonary fibrosis (IPF), 50% in sarcoidosis and 12.4% in cystic fibrosis. For patients with pulmonary hypertension, PGD was observed in 30.3% of patients with PPH and 29.3% of secondary pulmonary hypertension (SPH).

**Figure 4:**
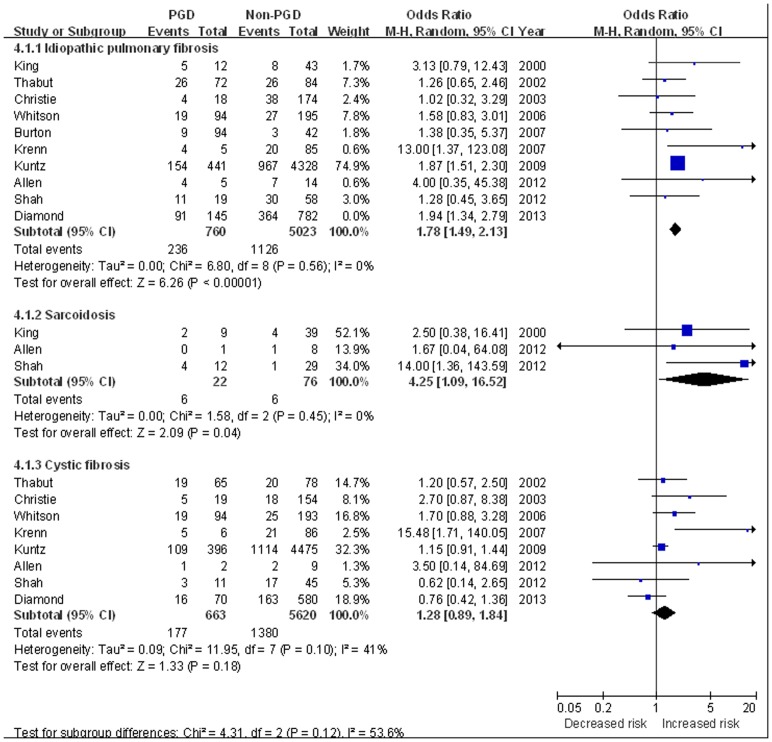
The influence of recipient pulmonary diagnosis on PGD. **COPD was used as the reference group.**

**Figure 5:**
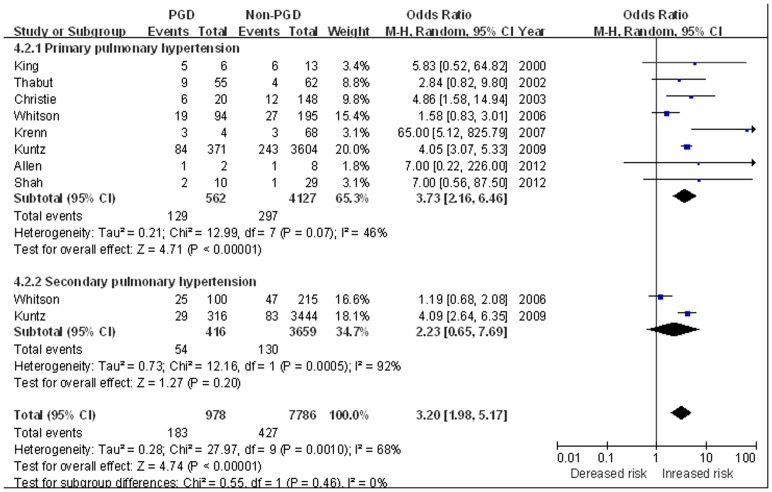
The influence of recipient pulmonary hypertension on PGD. **COPD was used as the reference group.**

Using COPD as the reference group (with the lowest incidence of PGD), IPF (OR 1.78, 95% CI 1.49 to 2.13, *P*<0.0001) [7]–[13], [16], [17], [19] and sarcoidosis (OR 4.25, 95% CI 1.09 to 16.52, *P* = 0.04) [7], [16]–[17] were both associated with increased risk of PGD; while cystic fibrosis was non-significantly associated with PGD development (OR 1.28, 95% CI 0.89 to 1.84, *P* = 0.18) [8]–[10], [12], [13], [16], [17], [19] (Figure 4). PPH was also significantly associated with PGD, with a 3.73-fold increased risk of PGD was observed (OR 3.73, 95%CI 2.16 to 6.46, *P*<0.001) [7]–[10], [12], [13], [16]–[17]; while unlike PPH, SPH did not confer an significantly increased risk of PGD (OR 2.23, 95%CI 0.65 to 7.69, *P* = 0.20) [10], [13] (Figure 5).

#### PAP

**Figure 6:**
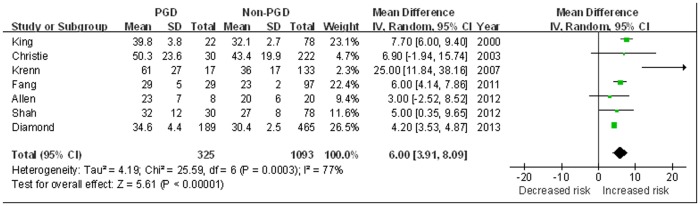
The influence of mean pulmonary artery pressures (PAP) on PGD.

There were 7 studies compared the mean PAP between patients with and without PGD (325 PGD patients and 1093 controls) [7], [9], [12], [15]–[17], [19]. Findings from the meta-analysis showed a significant higher PAP was observed in the PGD patients as compared with the controls (MD 6.00 mmHg, 95% CI 3.91 to 8.09 mmHg, *P*<0.0001). Statistical heterogeneity was observed among the studies (I^2^ = 77%, *P* = 0.0003) (Figure 6).

#### BLT vs. SLT

Eleven studies evaluated the impact of BLT vs. SLT on PGD development, including 4554 patients undergoing BLT and 5190 patients undergoing SLT [7]–[10], [12], [13], [15]–[19]. The pooled analysis showed the incidence of PGD was 14.5% in BLT recipients, compared to 13.8% in SLT recipients. Findings from the meta-analysis showed an insignificant association between the transplant type (SLT or BLT) and PGD (OR 1.10, 95% CI 0.97 to 1.24, *P* = 0.14). No statistically significant heterogeneity was observed between studies (I^2^ = 0%, *P* = 0.65).

#### BMI

Two studies evaluated the effect of BMI (as a continuous variable) on PGD [11], [14], including 155 patients with PGD and 147 controls. The pooled analysis of the 2 studies showed patients with PGD had a higher mean BMI level than controls (MD 1.20 kg/m^2^, 95% CI 0.13 to 2.27 kg/m^2^, *P* = 0.03). Other 2 studies investigated the impact of elevated BMI (BMI≥25 kg/m^2^) on PGD development [13], [19]. The incidence of PGD was 15.2% in the 3105 patients with elevated BMI, compared to 9.4% in the 5091 patients with normal BMI. Analysis of these studies showed a significant association between elevated BMI level and PGD (OR 1.83, 95% CI 1.26 to 2.64, *P* = 0.001).

#### CPB

Eleven studies evaluated the effect of CPB for PGD [7], [8], [10]–[12], [14]–[19]. PGD was found in 263 of 813 patients (32.3%) use of CPB compared to 490 of 1984 patients (24.7%) without CPB. The pooled analysis of these studies showed a 2.29-fold increased risk of PGD was present for patients requiring CPB (OR 2.29, 95%CI 1.43 to 3.65, *P* = 0.0005), with statistical heterogeneity among the studies (I^2^ = 69%, *P* = 0.0004).

#### Inhaled NO

Four studies investigated the influence of intra-operative use of inhaled NO on the occurrence of PGD [9], [14]–[16]. The incidence of PGD was 23.4% (50 of 214 patients) and 18.8% (59 of 314 patients) in patients with and without use of inhaled NO, respectively. Findings from this analysis suggested there was no significant association between intra-operative inhaled NO use and development of PGD (OR 1.09, 95% CI 0.68 to 1.74, *P* = 0.72). No statistical heterogeneity was observed between studies (I^2^ = 0%, *P* = 0.95).

#### Blood products transfusion

Three studies reported the amount of packed red blood cells (RBCs) and plasma used during the lung transplant procedure to evaluate the effect of intra-operative transfusion on PGD [14], [15], [19]. Findings from the meta-analysis showed a greater amount of packed RBCs and plasma transfused in patients with PGD compared with those without (RBCs: MD 341 ml, 95% CI 254 to 427 ml, *P*<0.001; plasma: MD 131 ml, 95% CI 71 to 191 ml, *P*<0.001). The χ^2^ test for heterogeneities were also non-significant (I^2^ = 0%, *P* = 0.84 and I^2^ = 44%, *P* = 0.17).

#### Mortality risk for PGD

**Figure 7:**
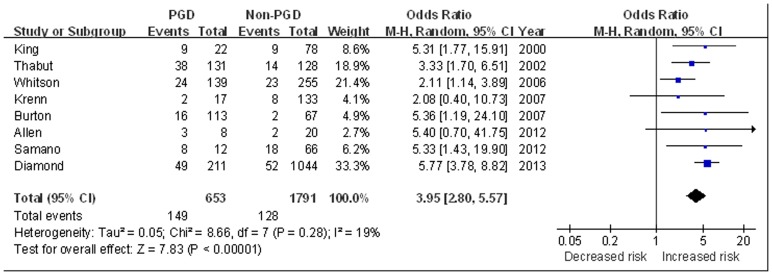
The influence of PGD on short-term mortality (mortality within 90 days).

The impact of PGD on mortality (within 90 days) was reported in 8 studies [7], [8], [10]–[12], [16], [18], [19]. All-cause mortality within 90 days was 22.8% for patients with PGD versus 7.1% for patients without. The pooled analysis suggested patients with PGD was associated with a nearly 4 fold increased risk of short-term mortality (OR 3.95, 95% CI 2.80 to 5.57, *P*<0.001) compared with those without PGD. There was no statistical heterogeneity among the studies (I^2^ = 19%, *P* = 0.28) (Figure 7).

#### Subgroup analysis according to the definitions for PGD

In the subgroup meta-analysis, we compared the associations between above risk factors and PGD in subsets of studies grouped by ISHLT PGD grade or other definitions (Table 3). The results showed no matter which definition was used in the original studies, no significant difference was observed in the effects of age, gender, race, pulmonary diagnosis, mPAP, BLT vs SLT, use of CPB, or inhaled NO in PGD development (*P* for subgroup difference >0.05).

**Table 3:** Subgroup analysis according to the definitions for PGD.

	No.of studies	Test for association		Test for subgroup difference	
		OR (95% CI)	*P*	I^2^	*P*
**Age**				0%	0.72
ISHLT	5	−1.33 (−5.09 to 2.43)	0.49		
Othe definitions	5	−0.58 (−2.17 to 1.10)	0.48		
**Female**				0%	0.33
ISHLT	8	1.21 (0.82 to 1.77)	0.33		
Othe definitions	4	1.50 (1.23 to 1.83)	<0.001		
**Race**					
African-American				36%	0.21
ISPGS	3	2.28 (1.55 to 3.36)	<0.001		
Othe definitions	3	1.37 (0.68 to 2.74)	0.38		
**Diagnosis**					
IPF				0%	0.58
ISHLT	5	1.88 (1.40 to 2.54)	<0.001		
Othe definitions	5	1.65 (1.14 to 2.38)	0.0009		
Cystic fibrosis				0%	0.71
ISHLT	5	1.41 (0.63 to 3.18)	0.41		
Othe definitions	3	1.20 (0.93 to 1.55)	0.16		
PPH				0%	0.61
ISHLT	5	6.58 (1.04 to 41.59)	<0.001		
Othe definitions	3	4.04 (3.12 to 5.24)	0.05		
**Mean PAP**				0%	0.61
ISHLT	4	5.80 (1.65 to 9.94)	0.006		
Othe definitions	3	6.93 (5.69 to 8.17)	<0.001		
**BLT vs SLT**				0%	0.73
ISHLT	6	1.06 (0.84 to 1.33)	0.63		
Othe definitions	5	1.11 (0.96 to 1.28)	0.15		
**CPB**				0%	0.77
ISHLT	7	2.31 (1.23 to 4.33)	0.009		
Othe definitions	4	2.62 (1.47 to 4.66)	0.001		
**Use of inhaled NO**				0%	0.69
ISHLT	2	1.22 (0.59 to 2.51)	0.60		
Othe definitions	2	1.00 (0.54 to 1.85)	0.99		

PGD, primary graft dysfunction; ISHLT, International Society for Heart and Lung Transplantation; IPF, idiopathic pulmonary fibrosis; PPH, primary pulmonary hypertension; PAP, pulmonary artery pressure; BLT, bilateral lung transplant; SLT, single lung transplant; CPB, cardiopulmonary bypass; NO, nitric oxide.

### Publication Bias

**Figure 8:**
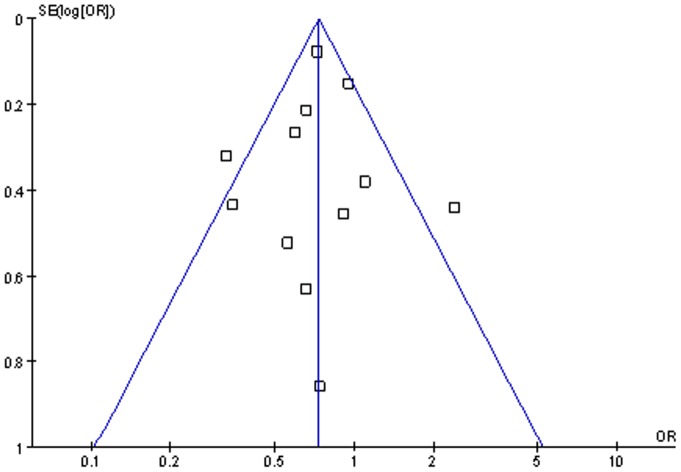
Funnel plot of the 12 studies evaluated the effect of the recipient gender on PGD.

We performed funnel plot analysis and Begg's test to assess publication bias. Funnel plot analysis was performed using the recipient gender as an index, the funnel plot of the 12 studies appeared to be symmetrical (Figure 8), and the Begg's test of funnel plot suggested no publication bias (*P* = 0.87). Also no publication bias was detected by Begg's test for other outcomes analysis (all *P*>0.05).

## Discussion

Despite the significant morbidity and mortality in patients with PGD after lung transplantation, the recipient related risk factors contributing to this devastating syndrome remain controversial. Our meta-analysis comprehensively reviewed 13 studies involving 10042 lung transplantation recipients which addressed the clinical risk factors for PGD. The results showed recipient female gender, African American race, preoperative diagnosis of IPF, sarcoidosis, or PPH, elevated mean PAP and BMI, use of CPB and blood products transfusion were significantly and consistently associated with development of PGD. All of these factors are likely to be measured and monitored in the primary care setting. To the best of our knowledge, this is the first systematic review on this topic.

Among baseline variables, we have demonstrated that female gender and African-American race had increased risk of PGD, which have not been validated in previous studies. Female gender has been associated with a higher risk of development of acute respiratory distress syndrome (ARDS) in the Ibuprofen in Sepsis Study Group [29], as well as in a cohort study of trauma patients [30]. Similarly, donor female gender was also shown to have an independent impact on PGD [9]. Possible mechanisms for these findings are unclear. Some theories for the differential outcome based on gender differences have been advocated, including immunity and tolerance theories [31] and the influence of gender hormones [32]. However, as of now few data have been published that evaluate the effect of gender on graft function and survival. Similarly, mechanisms for the observed worse outcome of African-American race remain speculative, but may reflect differences in vascular endothelium (such as expression of angiotensin-converting enzyme) [33], [34], which could potentially predispose African Americans to more severe ischemia reperfusion injury.

Elevated BMI was another risk factor for PGD in our meta-analysis. Prior studies have identified obesity as a risk factor for early mortality and increased intensive care unit stay after lung transplant [35], [36]. Technical difficulties of performing a lung transplant operation in obese recipients may increase risk of PGD. Other possible explanations may be obesity affects the milieu of cytokines produced by adipose tissue during ischemia-reperfusion, such as leptin [37], which has been shown to be increased in patients with acute lung injury and play a role in the development of acute lung injury in animal model [38]. In the study by Lederer et al, higher plasma leptin levels were associated with PGD after lung transplantation [39]. In addition, modulation of lung inflammation by other adipokines, such as resistin, adiponectin, which produced by macrophages recruited to hypertrophic and hypoxic adipose tissue, could also be responsible [39]–[41]. Future studies of adipokines in lung tissue or bronchoalveolar lavage fluid and examination of their roles in the development of PGD should be pursued.

In nearly all previous studies, diagnosis of PPH was the most significant risk factor for PGD [6], [10], [13], [19], and our findings further support this, showing both PPH and elevated mPAP were strongly associated with PGD after lung transplant. Possible explanations are not fully understood. In PPH, right ventricular dysfunction is universally present, and the hypertrophied, failing right ventricle is acutely afterload reduced at transplantation, resulting in increased shear stress on the formerly hypoxic pulmonary vascular endothelium. Shear stress leads to capillary leak and worse graft function [42], [43]. Christie et al showed diagnosis of PPH was even more strongly associated with an increased risk of PGD after adjustment for recipient PAP (adjusted RR = 9.24, *P* = 0.009) [9]. This implies it is the disease state of PPH that increases the risk, rather than just the presence or severity of pulmonary hypertension. Unlike PPH, our study suggested SPH did not confer an increased risk of PGD. In prior studies, the association between SPH and PGD was controversial and the conclusions were inconsistent [10], [13], [15], [18]. Fang et al demonstrated SPH in patients with IPF was independently associated with the development of PGD [15]. While for patients with CF, based on data from the ISHLT registry, no significant difference was observed in PGD incidence for patients with and without pulmonary hypertension [6]. These findings suggested that the association between pulmonary hypertension and PGD might depend on the underlying diagnoses to some extent. For studies included in this meta-analysis, SPH has been all-inclusive, regardless of cause [10], [13], [18]. Therefore, for further discussion, it is better to focus on the primary disease of SPH.

IPF was also identified as a risk factor with intermediate risk of PGD in our analysis. Previous observational studies reported patients undergoing transplantation for IPF had somewhat worse survival than for other indications, when matched on multiple variables [44], [45]. Possible explanations may be related to the pathogenesis of IPF. IPF carries a progressive course of pulmonary dysfunction that is inhibited, but not eliminated, by transplantation. Vasoactive mediators such as endothelin-1, platelet-derived growth factor, transforming growth factor-β, and fibroblast growth factor have all been implicated in the pathogenesis of IPF, and also contribute to the development of lung injury [46]. Moreover, IPF patients have a restrictive pattern of pulmonary disease with smaller-than-predicted total lung capacity. Shrinking lung volume may have caused irreversible damage to pulmonary mechanics by contracting the chest wall (remodeling). This relative “oversize” donor lung within smaller chest may lead to worse graft function [45], [47]. Nevertheless, at this point, reasons for the poor outcomes of IPF after transplantation remain elusive and warrant focused investigation.

The intra-operative use of CPB was another potential contributor to PGD in our meta-analysis. CPB causes a systemic, pro-inflammatory response with activation of cytokines, leukocytes and the complement cascade [48], [49]. Patients requiring CPB have been shown to have more radiographic infiltrates, worse immediate graft function, longer intubation, and ultimately, decreased survival [50], [51]. However, a notable difficulty in interpreting the data is the overall severity of the patient's illness or operative difficulty requiring the use of CPB. It is not possible to accurately differentiate planned use of CPB from emergent initiation intra-operatively because of deterioration in patient hemodynamics or oxygenation. As a result, independent of indication for CPB use, the association between PGD and CPB is still debatable. The type of transplant procedure (bilateral vs. single) was not identified as a significant risk factor for PGD in our study. Although the reported incidence of PGD was somewhat higher in BLT recipients, higher pre-transplant PAP and CPB use in BLT recipients likely confounded these results [5], [10].

The finding of blood products transfusion as a risk factor for PGD has been shown in recent multicenter studies and our meta-analysis confirmed this tendency [19], [52], but the exact relationship between the two processes is not yet clear. Blood products transfusion in-and-of-itself is associated with transfusion-related lung injury, which results in an ARDS-like picture similar to that seen with PGD [53]. The transfusion-related lung injury might accentuate any underlying mild ischemia/reperfusion injury, resulting in the onset of clinically significant PGD [53]. Nonetheless, the need for blood products administration has been shown to collinear with other PGD risk factors, including PPH and the use of CPB, and unmeasured operative characteristics may also lead to transfusion requirements [54]. Therefore dissecting the independence of the relationship between blood transfusion and PGD is difficult.

Inhaled NO has been investigated as a potential agent for the prevention of PGD, given its effects on pulmonary vasodilation and capillary integrity. Although our analysis did not support use of inhaled NO to be effective in PGD prevention, it may be beneficial in clinical settings of established PGD. Several reports and case series have shown improved outcomes with inhaled NO administration [55], [56]. However, there have also been studies that do not show efficacy in the setting of PGD [57]. Lack of randomized clinical trials showing survival benefit precludes widespread recommendation of inhaled NO for the treatment of PGD. Again, extrapolating from inhaled NO use in studies with ARDS, the beneficial effects of inhaled NO may be real, but also appear to be transient [58].

### Limitations of the review

Although we believe that the current meta-analysis provided useful information, some potential limitations should be addressed. Firstly, heterogeneity in our study is substantial and may be attributed to differences in type of patients, study era, operative practice, and definition of PGD. Definition of PGD is a major cause of heterogeneity, and with potential for misclassification bias. As the ISHLT PGD criteria were first published in 2005, studies performed before 2005 did not use standard defining criteria; even for the studies defined PGD based on the ISHLT guidelines, the PGD grades were retrospectively assigned to those patients enrolled before 2005. To clarify the heterogeneity, subgroup analyses were performed by dividing studies according to ISHLT or other definitions, and the results suggested our findings were not significantly affected by varying definitions. Secondly, our analysis was by necessity restricted to individual risk factors. Therefore, the distinct possibility exists that the strength of association may be weaker with a multi-factorial regression analysis; for instance, the individual effects of CPB, use of blood products, and elevated PAP cannot be delineated since they are often apparent in the same patients. In the present meta-analysis, it was not possible to adjust or stratify for potential confounders, which restricted us doing more detailed relevant analysis and obtaining more comprehensive results. Finally, given that a proportion of studies included are retrospective, a possibility of residual confounding variables by unmeasured factors cannot be eliminated. This provided associative, not causal, evidence and mandates caution when interpreting these results.

### Conclusion

Our systematic review and meta-analysis have identified several recipient-related risk factors for development of PGD, all of which are readily available in clinical settings. The identification of higher-risk recipients has great clinical relevance with respect to individual screening, risk factor modification, selective management aimed at prevention of PGD, and ultimately improves the outcomes of patients undergoing lung transplantation. Further research into the underlying mechanisms responsible for these associations should be advocated.

## Supporting Information

Checklist S1 The PRISMA Checklist for this Systematic Reviews and Meta-Analyses.
(DOC)
